# Supplementary material for: Re-order parameter of interacting thermodynamic magnets
Source: Nat Commun. 2024 Apr 17;15:3294. doi: 10.1038/s41467-024-47637-2 (PMC11024203; doi:10.1038/s41467-024-47637-2)
Supplement: Supplementary file 1 — Supplementary information [file 41467_2024_47637_MOESM1_ESM.pdf]

Supplementary Information for

Re-order parameter of interacting thermodynamic magnets

Byung Cheol Park[1,2,†], Howon Lee[3,†,‡], Sang Hyup Oh[3], Hyun Jun Shin[3], Young Jai Choi[3,*], Taewoo Ha[1,2,*]

[1]Sungkyunkwan University, Suwon 16419, Republic of Korea

[2]Center for Integrated Nanostructure Physics, Institute for Basic Science, Sungkyunkwan University, Suwon 16419, Republic of Korea

[3]Department of Physics, Yonsei University, Seoul 03722, Republic of Korea

[†] These authors equally contributed to this work.

* Corresponding authors: Y. J. C. (phylove@yonsei.ac.kr) & T. H. (bspha77@gmail.com)

[‡] Current affiliation: Center for Spintronics, Korea Institute of Science and Technology (KIST), Republic of Korea

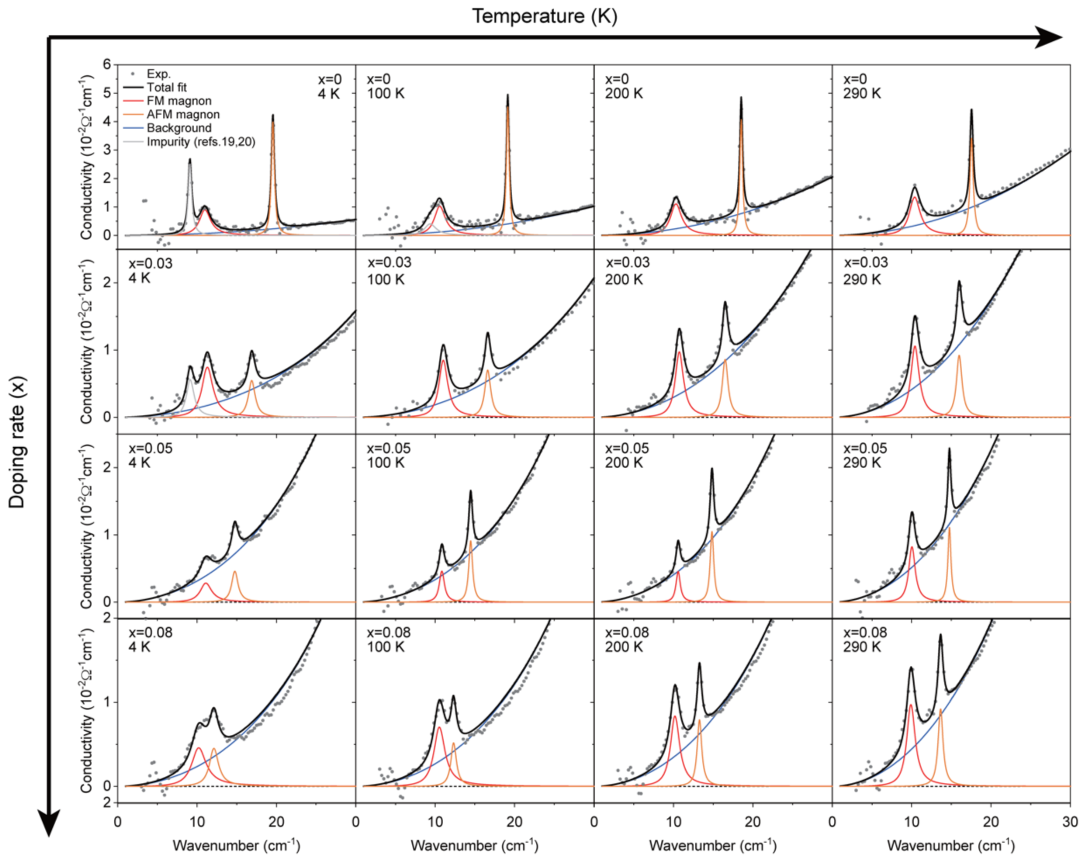

Supplementary Fig. 1| Results of model fitting to conductivity spectrum for lowly Mn-doped $YFe_{1-x}Mn_xO_3$ over $0 \leq x \leq 0.08$. Dots are experimental data, while black curves are total fitting lines. Red and orange lines indicate Lorentzians for magnons. FM (red) and AFM (orange) magnons are coexistent. Blue lines present an unknown background. Gray lines for x=0 at 4 K, 100 K, and x=0.03 at 4 K present the well-known impurity peaks (refs. 19, 20). See **Methods** for the fitting model.

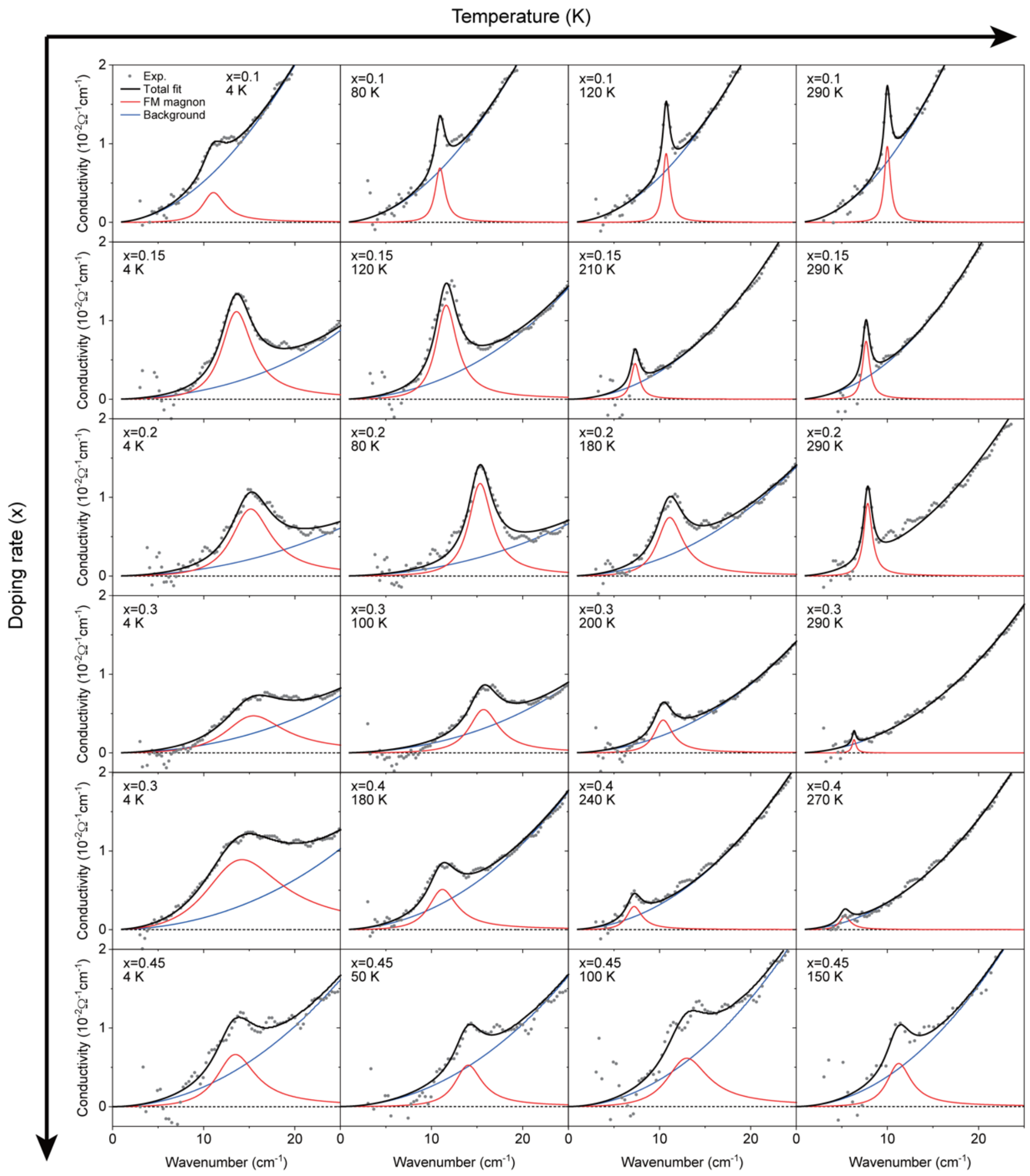

Supplementary Fig. 2| Results of model fitting to conductivity spectrum for highly Mn-doped $YFe_{1-x}Mn_xO_3$ over $0.1 \leq x \leq 0.45$. Dots are experimental data, while black curves are total fitting lines. Red lines indicate Lorentzians for magnons. AFM magnon disappears whereas FM magnon remains except the case x=0.45 at 4 K (FerriM magnon). Blue lines present an unknown background. See **Methods** for the fitting model.

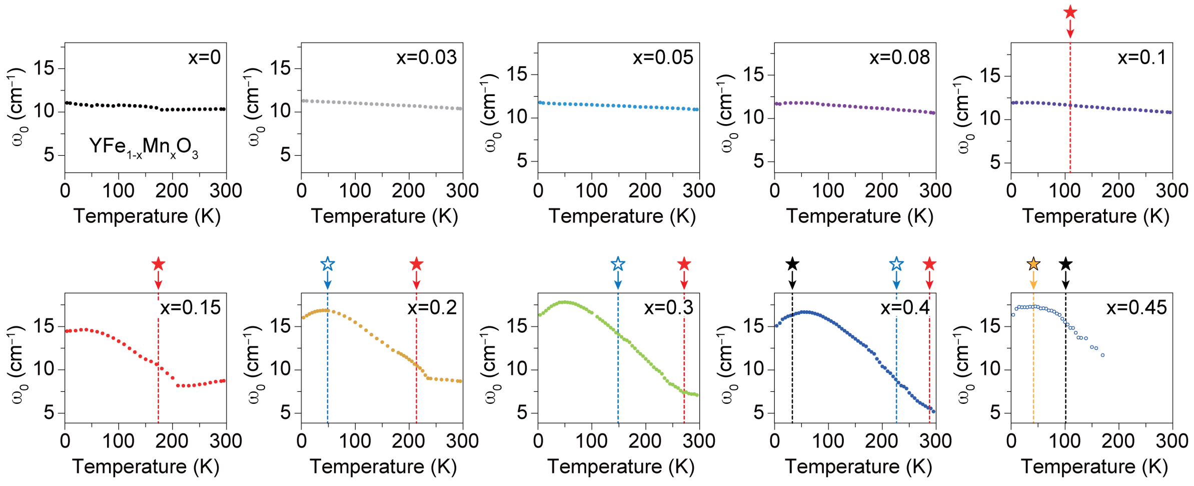

Supplementary Fig. 3| Another parameter, a precession frequency of magnon for Mn-doped $YFe_{1-x}Mn_xO_3$ over $0 \leq x \leq 0.45$. Stars in the plot correspond to the characteristic temperatures identified in Fig. 4 of the main text.

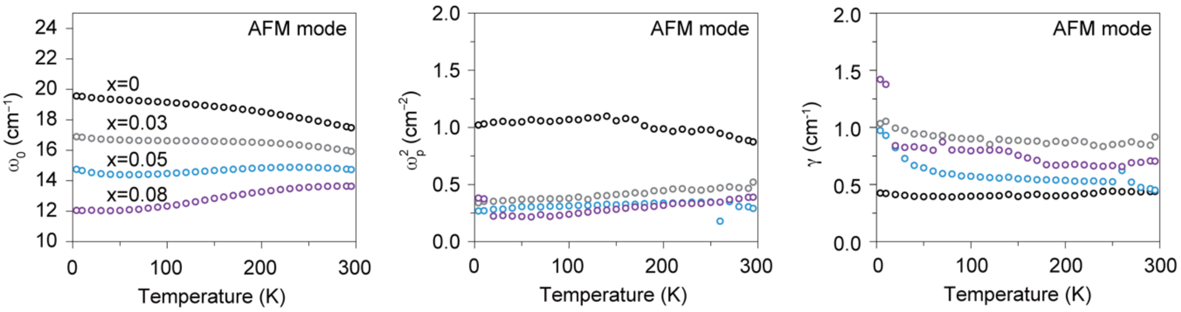

Supplementary Fig. 4| Magnon parameters of AFM mode for Mn-doped $YFe_{1-x}Mn_xO_3$ over 0 ≤ x ≤ 0.08. No transition is observed over 0 ≤ x ≤ 0.08. Above x=0.08, the AFM mode disappears.

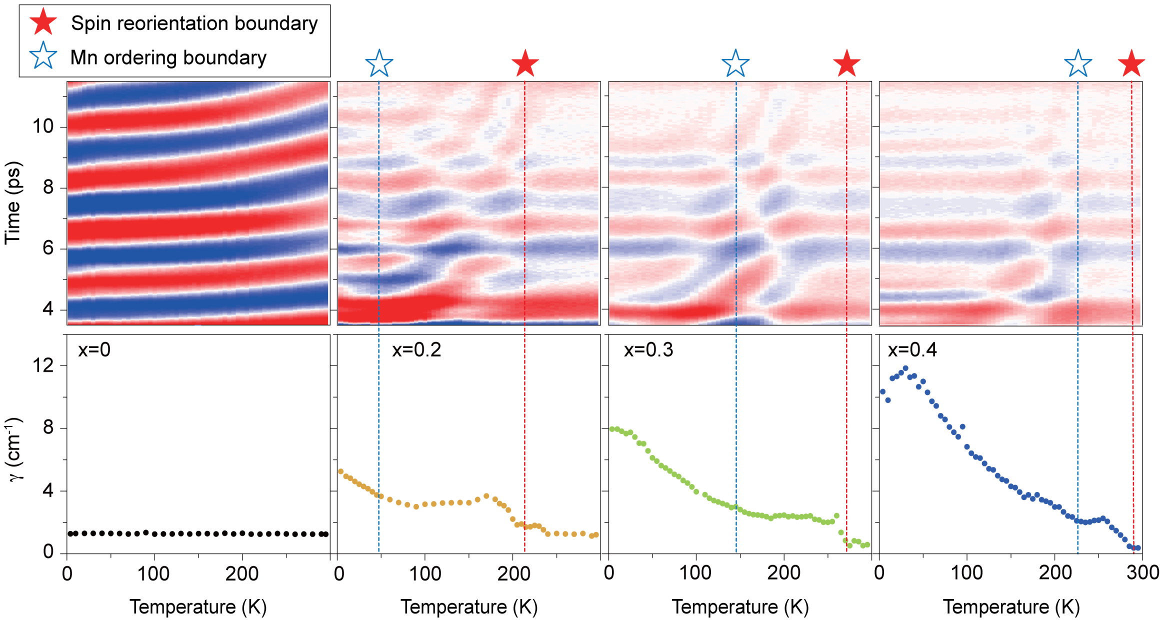

Supplementary Fig. 5| Correlation between time-domain and frequency-domain magnon parameters. This two-dimensional plot illustrates the oscillatory magnon emission signal, with temperature on the x-axis and time on the y-axis. The time-domain signal is compared with the re-order parameter (ω_p^2) and the auxiliary re-order parameter (γ). Stars in the plot correspond to the characteristic temperatures identified in Fig. 4 of the main text.

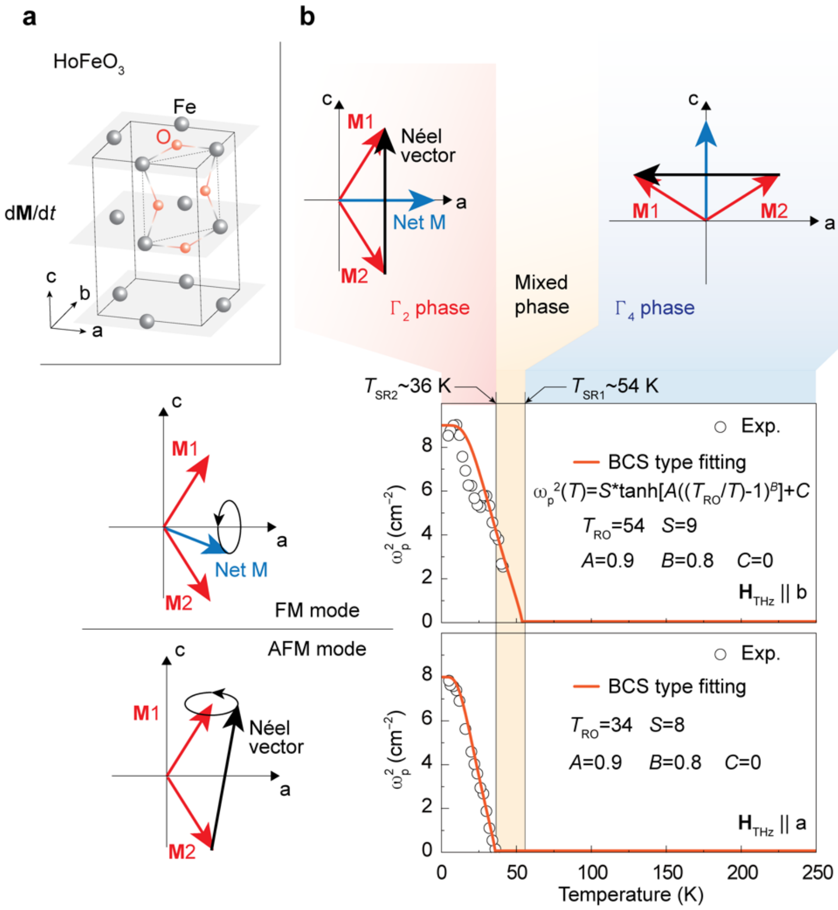

Supplementary Fig. 6| Re-order parameter for $HoFeO_3$. a, Crystal structure of $HoFeO_3$. **b**, Temperature dependent re-order parameter (ω_p^2, magnon density). Top panel for FM mode under H_{THz} || b-axis and bottom panel for AFM mode under H_{THz} || a-axis. Dots are experimental data and lines are BCS-type fitting results. Schematic illustrations are magnon modes (left figures) and the magnetic phases (top figures).
